# MiR-21-3p Inhibits Adipose Browning by Targeting FGFR1 and Aggravates Atrial Fibrosis in Diabetes

**DOI:** 10.1155/2021/9987219

**Published:** 2021-08-25

**Authors:** Jian-an Pan, Hao Lin, Jian-ying Yu, Hui-li Zhang, Jun-feng Zhang, Chang-qian Wang, Jun Gu

**Affiliations:** Department of Cardiology, Shanghai Ninth People's Hospital, Shanghai Jiaotong University School of Medicine, Shanghai, China

## Abstract

A relationship between excess epicardial adipose tissue (EAT) and the risk of atrial fibrillation (AF) has been reported. Browning of EAT may be a novel approach for the prevention or treatment of AF by attenuating atrial fibrosis. Previous studies have identified microRNA-21 (miR-21) as a regulatory factor in atrial fibrosis. The present study examined the role of different subtypes of miR-21 in adipose browning and atrial fibrosis under hyperglycemic conditions. Wild type and miR-21 knockout C57BL/6 mice were used to establish a diabetic model via intraperitoneal injection of streptozotocin. A coculture model of atrial fibroblasts and adipocytes was also established. We identified miR-21-3p as a key regulator that controls adipocyte browning and participates in atrial fibrosis under hyperglycemic conditions. Moreover, fibroblast growth factor receptor (FGFR) 1, a direct target of miR-21-3p, decreased in this setting and controlled adipose browning. Gain and loss-of-function experiments identified a regulatory pathway in adipocytes involving miR-21a-3p, FGFR1, FGF21, and PPAR*γ* that regulated adipocyte browning and participated in hyperglycemia-induced atrial fibrosis. Modulation of this signaling pathway may provide a therapeutic option for the prevention and treatment of atrial fibrosis or AF in DM.

## 1. Introduction

Despite many available treatments, diabetes mellitus (DM) remains a major public health problem that currently affects more than 425 million people worldwide [1]. DM can lead to many complications that affect the cardiovascular system, kidneys, retina, and nervous system due to long-term increased blood glucose levels [2]. Atrial fibrillation (AF) is the most common arrhythmia seen in clinical practice and is a major cause of morbidity and mortality as it increases the risk of abnormal hemodynamics and thromboembolism [3]. Several clinical studies [4, 5] have shown that type 1 and type 2 DM significantly promote AF via atrial structural and electrical remodeling. Extensive atrial fibrosis is a hallmark of AF and is considered to play an important role in initiating and perpetuating AF under hyperglycemic conditions [6].

Epicardial adipose tissue (EAT) is a metabolically active visceral fat reservoir that surrounds and infiltrates the myocardium and vessels and is a recognized source of proinflammatory mediators involved in the onset and development of various cardiovascular diseases (CVDs), such as coronary artery disease (CAD) and arrhythmias [7, 8]. Increased EAT volume or thickness is associated with DM and is correlated with left atrial enlargement, atrial fibrosis, and abnormal electrical conduction between cardiomyocytes, resulting in the onset of AF [9]. As with other adipose tissues, EAT comprises both white adipose tissue (WAT) and brown adipose tissue (BAT) and presents the potential for transdifferentiation from BAT to WAT, or vice versa [10]. Compared with less metabolically active WAT depots, BAT is characterized by an abundance of mitochondria, capillaries, and uncoupling protein-1 (UCP-1). BAT is thought to play a protective role against cardiometabolic dysfunction via nonshivering thermogenesis, and browning of WAT may present a novel approach for the prevention or treatment of CVD [11]. WAT browning is thought to be impaired in DM [12]. However, the role of adipose browning in DM-induced atrial fibrosis remains to be elucidated.

Fibroblast growth factor 21 (FGF21) has significant effects on energy balance, glucose metabolism, and lipid metabolism [13]. The main source of FGF21 was originally identified as the liver [14], and its role in inducing WAT browning is slowly emerging [15]. The biological roles of FGF21 are affected via downstream signaling pathways by binding to fibroblast growth factor receptors (FGFRs) and proliferator-activated receptor gamma (PPAR*γ*) activation [16]. FGFR1 has been shown to mediate FGF21 regulation in WAT.

MicroRNAs (miRNAs) are a class of endogenous, 20-22-nucleotide noncoding RNAs. Their main function is to regulate posttranscriptional regulation of target gene expression by binding to the 3′ untranslated region (3′-UTR) [17]. MiR-21 was previously shown to be associated with atrial fibrosis [18–20], but most of its mechanisms have focused on cardiomyocyte damage and fibroblast activation. Few reports have shown the relationship between miR-21 and adipose browning. In this work, we identified miR-21-3p (a subtype of the miR-21 family) as a key regulator that controls adipose browning and participates in atrial fibrosis under hyperglycemic conditions. Moreover, FGFR1 (a direct target of miR-21-3p) is decreased in these conditions and controls WAT to BAT differentiation via the FGFR1/FGF21/PPAR*γ* pathway.

## 2. Material and Methods

### 2.1. Animal Experiments

All animal procedures were performed according to the US National Institutes of Health Guide for the Care and Use of Laboratory Animals and were approved by the Institutional Animal Care and Use Committee at Shanghai Ninth People's Hospital of Shanghai Jiaotong University School of Medicine, China. All mice used for these studies had a C57BL/6J genetic background. Male WT and miR-21 KO mice, aged 8-10 weeks, were purchased from Shanghai Model Organisms Center (Shanghai, China) and used in the studies. Mice were randomly assigned to one of four groups: the saline-treated WT group, the streptozotocin- (STZ-) treated WT group, the saline-treated miR-21 KO group, and the STZ-treated miR-21 KO group, with each group containing six mice. WT and miR-21 KO mice were infused intraperitoneally with 150 mg/kg STZ or an equivalent volume of saline as a bolus, and the blood glucose level was checked after seven days. Mice with blood glucose levels of 16.7 mM were considered diabetic and used for experiments in the STZ-treated groups. The blood glucose levels of each group are shown in supplementary material Table S1. All mice were kept for 3 months. Both EAT and atrial tissues were collected under a stereomicroscope after removal of the pericardium and pericardial fat as previously described [21].

### 2.2. WT/miR-21 KO Mouse Preadipocyte Culture and Differentiation

Mouse preadipocyte isolation was performed as previously described [22]. In brief, subcutaneous adipose tissue in the groin was isolated under sterile conditions, washed twice in phosphate-buffered solution (PBS) supplemented with 1% penicillin/streptomycin, and minced into 1 mm^3^ pellets. Enzymatic digestion was performed with 2 mg/mL collagenase II (Worthington Biochemical, USA) at 37°C in a water bath for 1 h with gentle agitation and terminated using an equal volume of DMEM/F12 (Hyclone, USA) supplemented with 10% fetal bovine serum (FBS). The suspension was filtered using a 75 *μ*m nylon cell strainer and centrifuged at 2000 rpm for 5 min. After removing the lysed red blood cell solution, the suspension was centrifuged at 2000 rpm for 5 min and the preadipocytes were resuspended in DMEM/F12 supplemented with 10% FBS and diluted to a final concentration of 10^6^ cells/mL. The cells were cultured at 37°C in a humidified atmosphere containing 5% CO_2_. The preadipocytes isolated from WT and miR-21 KO mice were induced to differentiate into mature adipocytes as follows when reaching 80% confluence. Cells were incubated with 0.25 *μ*M dexamethasone, 0.5 mM 3-isobutyl-1-methylxanthine, and 10 *μ*g/mL insulin for 3 days, then they were rinsed thoroughly with culture media and incubated for an additional 3-4 days with 10 *μ*g/mL insulin. Differentiation was confirmed by morphological changes, including intracellular lipid droplet accumulation, by microscopic observation. Mature adipocytes were then treated with either low glucose (LG; 5.5 mM) and high glucose (HG; 30 mM) for 72 h.

### 2.3. Oil Red O Staining

Cultured adipocytes were washed with PBS and fixed with 4% formaldehyde at room temperature for 15 min. The cells were stained with the oil red O working solution containing 6 mL oil red O stock solution (5 g/L in isopropanol) and 4 mL double-distilled water for 20 min. After staining, the cells were washed with 60% isopropanol in PBS and observed using an Olympus TH4-200 microscope (Japan). Oil red O was quantified by measuring the optical density at 490 nm.

### 2.4. Cell Transfection and Infection

When the mouse preadipocytes reached 80% confluence, lentiviral vectors carrying miR-21-3p mimics, miR-21-5p mimics, miR-21-3p inhibitor, FGFR1 shRNA (5′-TGTAACCTCTTCTTCCTAGGTC-3′), pcDNA-FGFR1 (F: CCGCTCGAGGGCCATCGGGCTGGATAAG; R: CCGGAATTTGGGGACAGGGTTGGTAG), or negative control (NC) gene were added to the cells. After transfection for 24-48 h, RT-PCR was used to verify the transfection efficiency.

### 2.5. Isolation and Culture of Adult Mouse Atrial Fibroblasts

Adult mouse atrial fibroblasts were isolated as we previously described [23]. In brief, hearts from 8–10-week-old male C57BL/6 mice were rapidly excised and submerged in cold PBS. The atria were cut into pieces using a tissue chopper. The pieces were washed and cells were dissociated using 1 mg/mL collagenase II (Worthington Biochemical, USA). After incubation for 30 min at 37°C, tissue pieces were discarded, and the supernatant, containing the isolated cells, was centrifuged at 1500 rpm for 5 min. Cells were resuspended in DMEM containing 10% FBS, and the resulting cell mixture was preplated and incubated in a 5% CO_2_ incubator at 37°C for 90 min. After removing the myocyte-enriched medium, DMEM was added to the preplated fibroblasts and cultured for 3-4 days before being passaged. The cultured cells were characterized as previously described [23].

### 2.6. Coculture of Atrial Fibroblasts and Adipocytes

Atrial fibroblasts and mature adipocytes were cocultured using transwell inserts with a 0.4 *μ*m porous membrane, which separated the adipocytes (5.0 × 10^5^ cells/well) in the lower chamber from the fibroblasts (5.0 × 10^5^ cells/well) in the upper chamber. Each cell type was grown independently on transwell plates. A hanging insert was constructed with a membrane with pores large enough to allow the passage of small molecules, yet small enough to prevent the passage of even the most motile cell types. Following coculture for 72 h after the incubation period, biomarkers for adipocyte browning and fibrosis-related gene expressions were measured in adipocytes and fibroblasts, respectively. Cocultured atrial fibroblasts and adipocytes were treated with LG (5.5 mM) or HG (30 mM) for 72 h in a humidified atmosphere containing 5% CO_2_ at 37°C.

### 2.7. Histology, Immunohistochemical Staining, and Immunofluorescence Staining

EAT or atrial tissues from mice were fixed with 10% phosphate-buffered formalin for 24 h. Fixed tissues were then paraffin-embedded and serially sectioned using a microtome (4 *μ*m thickness). The extent of interstitial fibrosis was evaluated by Masson's staining. Immunohistochemical staining for UCP-1 in EAT sections was also performed. Immunohistochemical staining was performed for collagen I and collagen III in vitro cultured atrial fibroblasts. Immunofluorescence staining of UCP-1 was performed for in vitro cultured adipocytes. Images were acquired and analyzed using Image J.

### 2.8. Western Blot Analysis

Protein was extracted from EAT, atrial tissue, adipocytes, and atrial fibroblasts and then subjected to sodium dodecyl sulfate-polyacrylamide gel electrophoresis and transferred to a polyvinylidene difluoride membrane. Membranes were blocked with Tris-buffered saline with 0.1% Tween containing 5% milk and incubated overnight at 4°C with the following primary antibodies as indicated: GAPDH mouse polyclonal antibody (1 : 5000, 60004-1-Ig, Proteintech), TGF-*β*1 rabbit polyclonal antibody (1 : 1000, ab92486, AbCam), connective tissue growth factor (CTGF) rabbit polyclonal antibody (1 : 1000, ab6992, AbCam), collagen I rabbit polyclonal antibody (1 : 1000, ab34710, AbCam), collagen III rabbit polyclonal antibody (1 : 1000, ab7778, AbCam), UCP-1 rabbit monoclonal antibody (1 : 1000, ab209483, AbCam), FGFR1 rabbit polyclonal antibody (1 : 1000, ab10646, AbCam), FGF21 rabbit monoclonal antibody (1 : 1000, ab171941, AbCam), and PPAR*γ* rabbit polyclonal antibody (1 : 1000, ab45036, AbCam). Membranes were then incubated with horseradish peroxidase-conjugated secondary antibodies and visualized using an enhanced chemiluminescence system. Densitometric analysis was performed using Scion Image software (USA).

### 2.9. RNA Preparation and Analysis

Total RNA was extracted from adipocytes using the TRIzol Reagent (Invitrogen, USA) and reverse-transcribed into cDNA using the PrimeScript™ RT Reagent Kit (Takara, Japan). Next, the cDNA was quantitatively amplified using TB Green Premix Ex Taq II (Takara, Japan). Real-time PCR was performed in triplicate using an Applied Biosystems 6Flex. The sequences of the forward and reverse primers used for amplification are shown in supplementary material Table S2. The gene expression of FGFR1 was presented relative to that of GAPDH, and relative miR-21-3p and miR-21-5p expression were normalized to that of U6 small nuclear RNA (snRNA) by the delta-delta Ct method.

### 2.10. ELISA Measurement of Inflammatory Cytokines

Murine tumor necrosis factor alpha (TNF-*α*), interleukin 6 (IL-6), and monocyte chemoattractant protein 1 (MCP-1) levels in coculture supernatants were measured by ELISA (TNF-*α*, ab208348; IL-6, ab100712; MCP-1, ab246547; AbCam, USA) according to the manufacturer's instructions.

### 2.11. Statistical Analysis

Statistical analysis was performed by using SPSS 22.0 software. All arithmetic data were presented as mean ± SD of least three independent experiments. The two-tailed *t*-test was used to analyze the differences between 2 groups, and one-way ANOVA followed by Bonferroni's post hoc test was used to analyze the differences among 3 or more groups. All *P* value < 0.05 was considered statistically significant.

## 3. Results

### 3.1. miR-21 Participated in Atrial Fibrosis and EAT Browning in the Mouse DM Model

**Figure 1:**
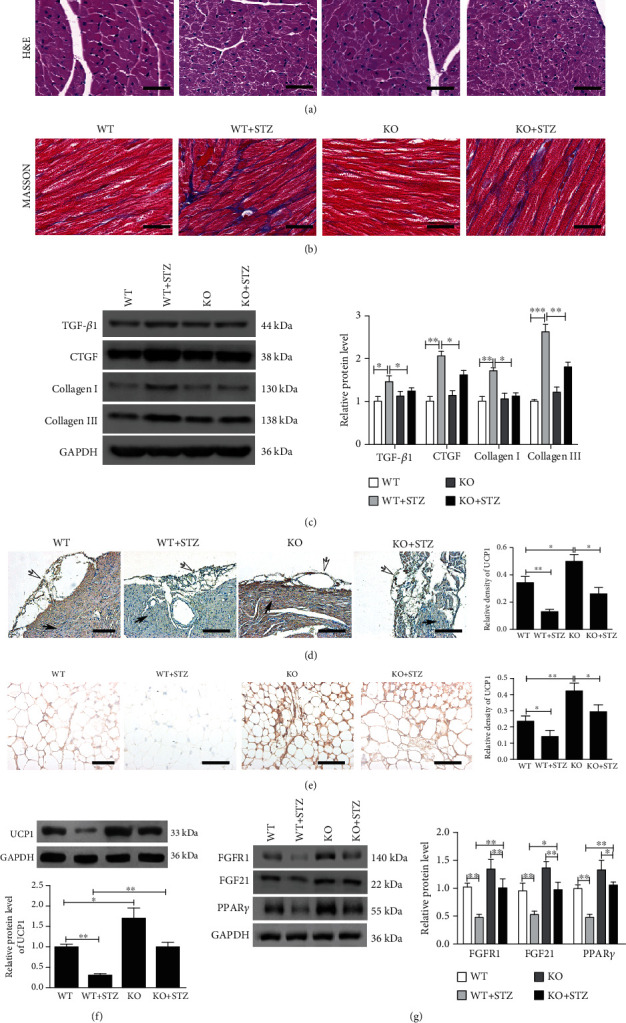
Atrial fibrosis and browning of pericardial adipose tissue were analyzed 3 months after the modeling. (a) H&E staining of atrial myocardial tissue samples, scale bar = 50 *μ*m. (b) Masson's staining of atrial myocardial tissue samples, scale bar = 50 *μ*m. (c) TGF-*β*1, CTGF, collagen I, and collagen III expressions in atrial myocardial tissue were analyzed by western blot and quantified by Image J. (d) Representative images and quantification of UCP1 expression were analyzed by immunohistochemistry in sections containing both pericardial adipose tissue and atrial myocardium from the hearts of mice. Hollow arrows indicate pericardial adipose tissue; solid arrows indicate atrial myocardium. Scale bar = 200 *μ*m. (e) Representative images and quantification of UCP1 expression were analyzed by immunohistochemistry in collected pericardial adipose tissue, scale bar = 50 *μ*m. (f) UCP1 expression in collected pericardial adipose tissue was analyzed by western blot and quantified by Image J. (g) FGFR1, FGF21, and PPAR*γ* expression in collected pericardial adipose tissue were analyzed by western blot and quantified by Image J. The data are presented as the mean ± SD of six mice per group. ^∗^*P* < 0.05, ^∗∗^*P* < 0.01, and ^∗∗∗^*P* < 0.001.

Compared with the control group, DM induced significant atrial interstitial collagen deposition, which was significantly attenuated by miR-21 KO (Figures 1(a) and 1(b)). Furthermore, DM induced increased expression of fibrosis-related proteins, such as TGF-*β*1, CTGF, collagen I, and collagen III in atrial tissue, which were also markedly alleviated by miR-21 KO (Figure 1(c)). Interestingly, hyperglycemic conditions led to a decreased expression of the adipose browning biomarker, UCP-1, which was significantly reversed in miR-21 KO mice (Figures 1(d)–1(f)). Furthermore, evaluation of WAT browning-associated proteins revealed decreased expression of FGFR1, FGF21, and PPAR*γ* in the diabetic model that could be partially reversed by miR-21 KO (Figure 1(g)). These results suggested that miR-21 is involved in atrial fibrosis in diabetes, perhaps related to its effect on EAT browning.

### 3.2. miR-21-3p Played a Major Role in Adipose Browning

**Figure 2:**
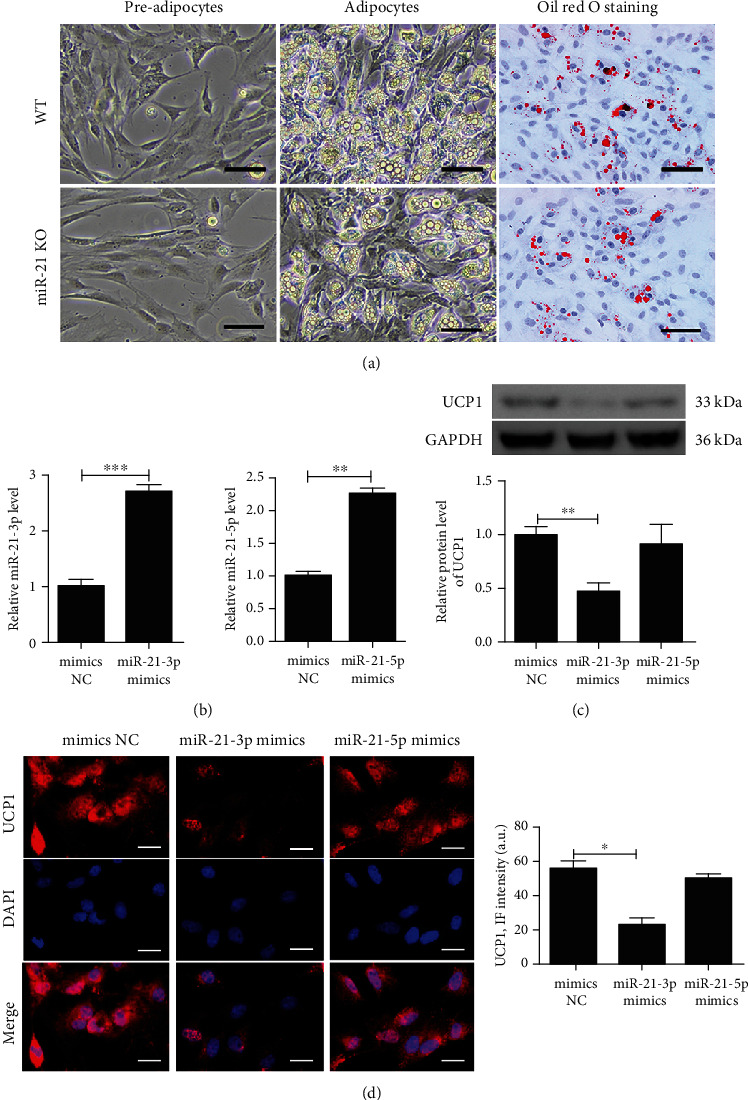
MiR-21-3p plays a major role in adipose browning. (a) Adipocytic differentiation of preadipocytes isolated from WT or miR-21 KO mice and oil red O staining of mature adipocytes after differentiation induction, scale bar = 50 *μ*m. Preadipocytes from miR-21 KO mice were induced to differentiate into mature adipocytes and then transfected with mimics NC, miR-21a-3p mimics, or miR-21a-5p mimics. (b) The relative miR-21-3p and miR-21-5p expressions were detected by qRT-PCR in mature adipocytes. (c) UCP1 expression was analyzed by western blot and quantified by Scion Image software. (d) Representative images and quantification of UCP1 expression were detected by immunofluorescence in mature adipocytes, scale bar = 50 *μ*m. UCP1 (red) is shown; nuclei are stained by DAPI (blue). The data are presented as the mean ± SD of three independent experiments. ^∗^*P* < 0.05, ^∗∗^*P* < 0.01, and ^∗∗∗^*P* < 0.001.

We investigated the expression of different miR-21 subtypes in EAT and atrial tissues of WT mice. The result showed that both in EAT and atrial tissues, the expression of miR-21-3p, instead of miR-21-5p, was significantly increased after STZ treatment. (Figure S1). To investigate the role of miR-21-3p in EAT, preadipocytes were isolated from miR-21 KO mice and differentiation was induced into mature adipocytes. Oil red O staining of mature adipocytes is shown in Figure 2(a). And the mature adipocytes were transfected with mimics NC, miR-21-3p mimics, or miR-21-5p mimics to achieve overexpression of the corresponding miR-21 subtype (Figure 2(b)). Western blot and immunofluorescence showed that UCP-1 expression was significantly inhibited by transfection with miR-21-3p mimics, but not with miR-21-5p mimics (Figures 2(c) and 2(d)), indicating that miR-21-3p is playing a major role in adipose browning, rather than miR-21-5p.

### 3.3. miR-21-3p Inhibited Adipose Browning to Promote Hyperglycemia-Induced Atrial Fibrosis

**Figure 3:**
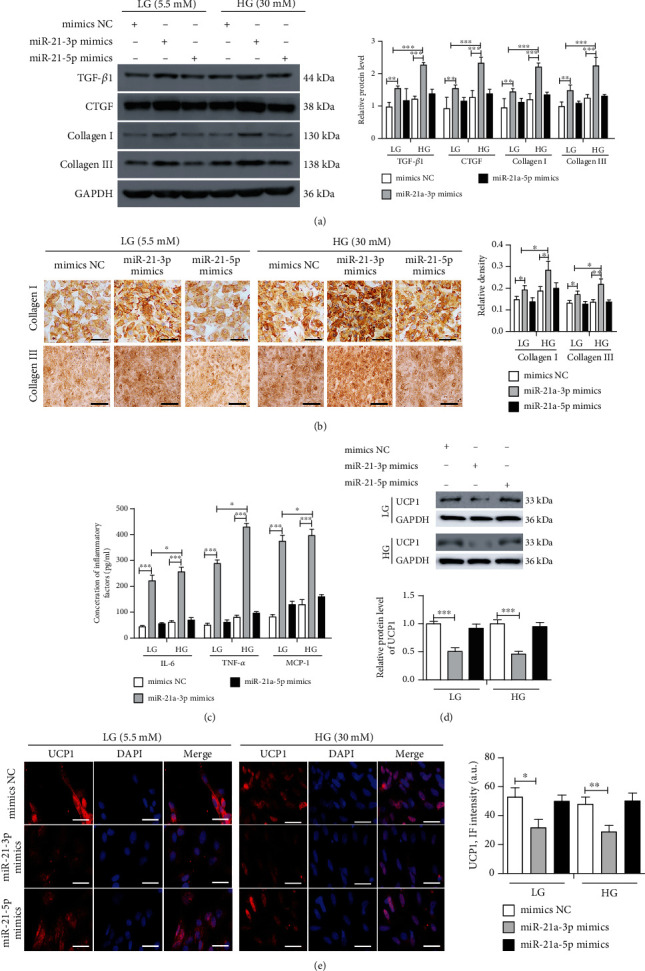
MiR-21-3p inhibits adipose browning to promote HG-induced myocardial fibrosis. Preadipocytes from miR-21 KO mice were induced to differentiate into mature adipocytes and then transfected with mimics NC, miR-21-3p mimics, or miR-21-5p mimics. Mature adipocyte coculture with atrial fibroblasts from WT mice treated with LG (5.5 mM) and HG (30 mM) for 72 h. (a) TGF-*β*1, CTGF, collagen I, and collagen III expressions in atrial fibroblasts were analyzed by western blot and quantified by Scion Image software. (b) Representative images and quantification of collagen I and collagen III expressions in atrial fibroblasts were analyzed by immunohistochemistry, scale bar = 100 *μ*m. (c) ELISA was used to analyze IL-6, TNF-*α*, and MCP-1 concentrations in coculture supernatants. (d) Western blot was used to analyze UCP1 expression in mature adipocytes and quantified by Scion Image software. (e) Representative images and quantification of UCP1 expression in mature adipocytes were detected by immunofluorescence, scale bar = 50 *μ*m. UCP1 (red) was shown; nuclei were stained by DAPI (blue). The data are presented as the mean ± SD of three independent experiments. ^∗^*P* < 0.05, ^∗∗^*P* < 0.01, and ^∗∗∗^*P* < 0.001.

The preadipocytes from miR-21 KO mice were induced to differentiate into mature adipocytes, and then were transfected with mimics NC, miR-21-3p mimics, or miR-21-5p mimics. Fully differentiated adipocytes were cocultured with atrial fibroblasts from WT mice and treated with either LG (5.5 mM) or HG (30 mM) for 72 h. We observed that hyperglycemic conditions induced a significantly higher expression of fibrosis-related proteins in fibroblasts (Figures 3(a) and 3(b)). Under the same hyperglycemic conditions, miR-21-3p-transfected adipocytes showed an upregulated expression of fibrotic proteins in fibroblasts compared with cell adipocytes transfected with mimics NC or miR-21-5p mimics (Figures 3(a) and 3(b)). We also examined inflammatory factors in coculture supernatants, including TNF*α*, IL-6, and MCP-1, and found that these inflammatory factors were markedly increased by transfection with miR-21-3p mimics in adipocytes compared with cells transfected with mimics NC or miR-21-5p mimics under both LG and HG conditions (Figure 3(c)). The same trend was also observed for the expression of the adipose browning biomarker, UCP1 (Figures 3(d) and 3(e)). These results indicated that miR-21-3p may be involved in adipose browning and participate in hyperglycemia-induced atrial fibrosis.

### 3.4. MiR-21-3p Regulated the FGFR1/FGF21/PPAR*γ* Pathway to Participate in Adipose Browning by Targeting FGFR1

**Figure 4:**
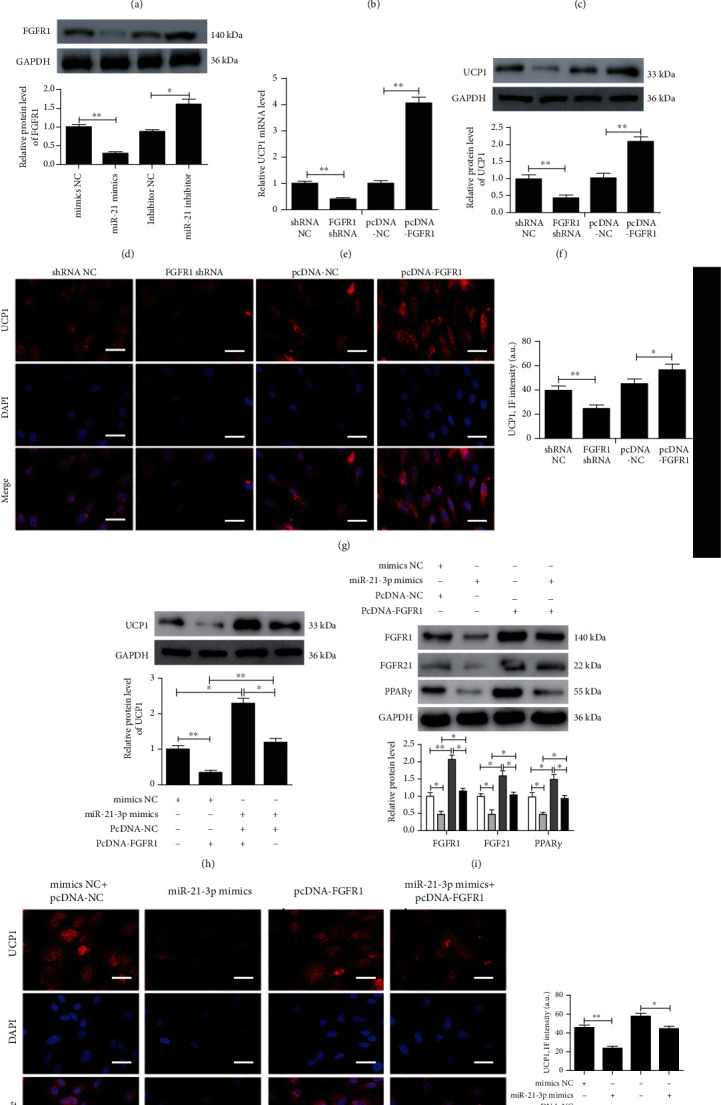
MiR-21-3p regulated the FGFR1/FGF21/PPAR*γ* pathway to participate in adipose browning by targeting FGFR1. (a) Putative miR-21-3p binding sites in the FGFR1 3′-UTR. (b) A luciferase reporter plasmid containing wild-type or mutant FGFR1 was cotransfected into 293 cells with miR-21-3p mimics or control mimics. Luciferase activity was determined at 48 h after transfection. Preadipocytes extracted from WT mice were induced to differentiate into mature adipocytes and then transfected with mimics NC, miR-21-3p mimics, inhibitor NC, or miR-21-3p inhibitor. (c, d) qRT-PCR and western blot were determined to analyze FGFR1 expression. Preadipocytes extracted from WT mice were induced to differentiate into mature adipocytes and then transfected with shRNA NC, FGFR1 shRNA, pcDNA-NC, or pcDNA-FGFR1. (e) The relative UCP1 expression was detected by qRT-PCR. (f) Western blot was used to analyze UCP1 expression and quantified by Scion Image software. (g) Representative images and quantification of UCP1 expression were detected by immunofluorescence, scale bar = 50 *μ*m. UCP1 (red) was shown; nuclei were stained by DAPI (blue). Preadipocytes from miR-21 KO mice were induced to differentiate into mature adipocytes and then transfected with mimics NC+pcDNA-NC, miR-21-3p mimics, pcDNA-FGFR1, or miR-21-3p mimics+pcDNA-FGFR1. (h, i) Western blot was used to analyze UCP1, FGFR1, FGF21, and PPAR*γ* expression and quantified by Scion Image software. (j) Representative images and quantification of UCP1 expression were detected by immunofluorescence, scale bar = 50 *μ*m. UCP1 (red) was shown; nuclei were stained by DAPI (blue). The data are presented as the mean ± SD of three independent experiments. ^∗^*P* < 0.05, ^∗∗^*P* < 0.01, and ^∗∗∗^*P* < 0.001.

Analysis of the Mirdb (http://www.mirdb.org) and TargetScan (http://www.targetscan.org) databases showed that FGFR1 was a potential target gene of miR-21-3p. The matching positions for miR-21-3p within the 3′-UTR of the targeted FGFR1 mRNA are shown in Figure 4(a). The dual-luciferase assay revealed that miR-21-3p reduced luciferase activity (Figure 4(b)). Preadipocytes from WT mice were induced to differentiate into mature adipocytes (Figure 3(a)) and then transfected with mimics NC, miR-21-3p mimics, inhibitor NC, or miR-21-3p inhibitor. FGFR1 mRNA and protein expression was decreased in adipocytes overexpressing miR-21-3p and increased in adipocytes silencing miR-21-3p (Figures 4(c) and 4(d)). The mature adipocytes were transfected with shRNA NC, FGFR1 shRNA, pcDNA-NC, or pcDNA-FGFR1. UCP1 expression was decreased following FGFR1 shRNA transfection and was upregulated after pcDNA-FGFR1 transfection at both mRNA and protein (Figures 4(e)–4(g)). Preadipocytes from miR-21 KO mice were induced to differentiate into mature adipocytes and then transfected with mimics NC+pcDNA-NC, miR-21-3p mimics, pcDNA-FGFR1, or miR-21-3p mimics+pcDNA-FGFR1. The results showed that miR-21-3p efficiently suppressed UCP-1 expression in adipocytes by inhibiting FGFR1 via the FGFR1/FGF21/PPAR*γ* pathway, which was partially reversed by upregulation of FGFR1 via pcDNA-FGFR1 transfection (Figures 4(h)–4(j)).

### 3.5. MiR-21-3p Regulated the Adipose Browning-Associated Pathway FGFR1/FGF21/PPAR*γ* to Promote Hyperglycemia-Induced Atrial Fibrosis by Targeting FGFR1

**Figure 5:**
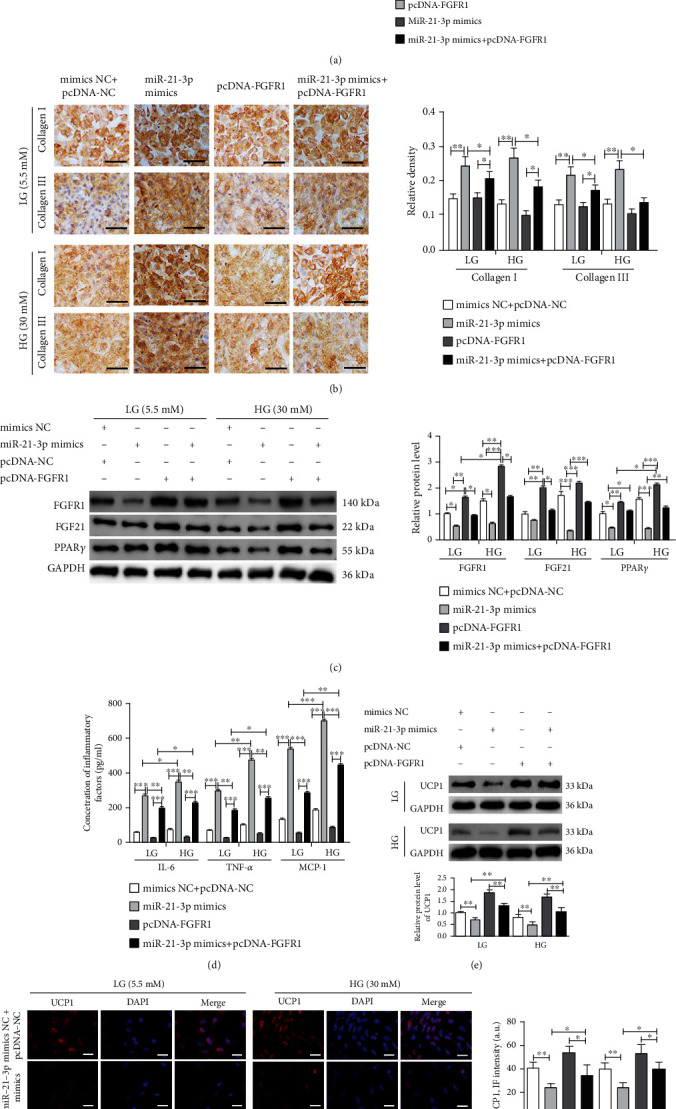
MiR-21-3p regulates the adipose browning-associated pathway FGFR1/FGF21/PPAR*γ* to promote HG-induced myocardial fibrosis by targeting FGFR1. Preadipocytes from miR-21 KO mice were induced to differentiate into mature adipocytes, and were then transfected with mimics NC+pcDNA-NC, miR-21-3p mimics, pcDNA-FGFR1, or miR-21-3p mimics+pcDNA-FGFR1. Mature adipocyte coculture with atrial fibroblasts from WT mice treated with LG (5.5 mM) and HG (30 mM) for 72 h. (a) TGF-*β*1, CTGF, collagen I, and collagen III expressions in atrial fibroblasts were analyzed by western blot and quantified by Scion Image software. (b) Representative images and quantification of collagen I and collagen III expressions in atrial fibroblasts were analyzed by immunohistochemistry, scale bar = 100 *μ*m. (c) Western blot was used to analyze FGFR1, FGF21, and PPAR*γ* expression in mature adipocytes, and the results were quantified by Scion Image software. (d) ELISA was used to analyze TNF-*α*, IL-6, and MCP-1 concentration in coculture supernatants. (e) Western blot was used to analyze UCP1 expression in mature adipocytes, and the results were quantified by Scion Image software. (f) Representative images and quantification of UCP1 expression in mature adipocytes were detected by immunofluorescence, scale bar = 50 *μ*m. UCP1 (red) was shown; nuclei were stained by DAPI (blue). The data are presented as the mean ± SD of three independent experiments. ^∗^*P* < 0.05, ^∗∗^*P* < 0.01, and ^∗∗∗^*P* < 0.001.

The preadipocytes from miR-21 KO mice were induced to differentiate into mature adipocytes, and then were transfected with mimics NC+pcDNA-NC, miR-21-3p mimics, pcDNA-FGFR1, or miR-21-3p mimics+pcDNA-FGFR1. Mature adipocytes were cocultured with atrial fibroblasts from WT mice and were treated with LG (5.5 mM) and HG (30 mM) for 72 h. The results showed that the HG treatment led to an efficiently increased expression of fibrotic proteins in fibroblasts compared with LG conditions (Figures 5(a) and 5(b)). Under the same conditions, transfection of adipocytes with miR-21-3p mimics resulted in higher expression of fibrotic protein compared with mimics NC+pcDNA-NC, and transfection with pcDNA-FGFR1 could partially reverse this phenomenon (Figures 5(a) and 5(b)).

Western blotting was also used to analyze FGFR1, FGF21, and PPAR*γ* expression in adipocytes in our coculture model. MiR-21-3p mimic transfection in adipocytes led to inhibition of adipose browning-associated proteins under HG conditions compared with LG conditions. Furthermore, under HG conditions, transfection with miR-21-3p mimics into adipocytes resulted in lower expression of adipose browning-associated proteins compared with transfection with mimics NC+pcDNA-NC, and transfection with pcDNA-FGFR1 could partially reverse this phenomenon (Figure 5(c)). Three inflammatory factors (TNF*α*, IL-6, and MCP-1) were markedly increased by miR-21-3p mimic transfection compared with mimics NC+ pcDNA-NC under both LG and HG conditions, and transfection with pcDNA-FGFR1 could partially reverse this phenomenon (Figure 5(d)). Finally, the adipose browning biomarker, UCP1, was markedly inhibited by transfection with miR-21-3p mimics, and pcDNA-FGFR1 transfection could partially reverse this phenomenon (Figures 5(e) and 5(f)).

## 4. Discussion

Studies have suggested that diabetes is a strong, independent risk factor for AF [6, 24]. While the underlying mechanisms by which diabetes increases susceptibility to AF remain unclear, they are thought to be associated with electrical and structural remodeling of the atria [25, 26]. Extensive atrial fibrosis resulting from hyperglycemia is thought to play a key role in both initiating and perpetuating AF, as an increase of collagen deposition in the atria can cause abnormal conduction of signals, rupture of propagating waves, and even reentry [27]. Recent studies have shown a higher volume or thickness of EAT in patients with AF, particularly in those with nonparoxysmal AF [28, 29]. EAT comprises WAT and BAT. BAT is the primary site of nonshivering thermogenesis and is, therefore, a relevant site for adaptive energy expenditure processes. Compared with WAT, BAT improves insulin sensitivity, glucose tolerance, lipid homeostasis, and protects against the pathogenesis of CVD [11, 30]. It was recently shown that adipose tissue has remarkable plasticity in terms of its contents of white and brown adipocyte content. Modulation of cardiac and vascular adipose tissue to increase the proportion of thermogenic brown or beige adipocytes could be a viable way to improve local inflammation and reduce cardiovascular risk [11, 30]. However, impaired WAT browning potential is observed under diabetic or hyperglycemic conditions [12], which may aggregate the pathogenesis of AF. Consistent with previous studies, our experiments indicated that hyperglycemia inhibited the biomarkers of BAT in mouse EAT as well as in adipocytes cultured in vitro, suggesting that hyperglycemia may decrease the process of adipose browning.

The mechanisms of adipose browning and its role in DM-induced atrial fibrosis remain unclear. FGF21 has been shown to have a beneficial effect on metabolism and energy balance by enhancing fatty acid oxidation during prolonged fasting and by promoting WAT browning [31, 32]. A recent clinical study showed that cold exposure increased circulating levels of the fat browning activators, FGF21, and irisin, and that treatment with either of these endocrine regulators upregulated browning genes and promoted thermogenesis [33]. Mechanistically, FGF21 activates cell signaling by binding to a heteromeric cell surface receptor tyrosine kinase complex composed of *β*-klotho and FGFR1 [32]. Both *β*-klotho and FGFR1 are abundantly expressed in WAT, where FGF21-regulated genes are involved in a variety of metabolic processes including lipogenesis, lipolysis, fatty acid oxidation, and WAT browning [32]. Furthermore, PPAR*γ*, which is a member of the nuclear receptor family of ligand-activated transcription factors, is also required for adipocyte differentiation. Treatment with PPAR*γ* agonists was shown to induce adipose browning and likely contributed to the increase in lipid turnover [34]. FGF21 was reported to be a key mediator of the physiological and pharmacological actions of PPAR*γ* in WAT [32]. On the other hand, FGF21 was previously shown to be induced by PPAR*γ* agonists in WAT and cooperate with rosiglitazone to promote differentiation of 3T3-L1 adipocytes [35, 36]. Obese, insulin-resistant mice lacking FGF21 are refractory to the actions of rosiglitazone, including both beneficial and adverse effects [32]. Therefore, we conclude that the actions of FGF21 and PPAR*γ* are fundamentally intertwined, and propose a feed-forward regulatory model in WAT [37]. FGFR1/FGF21/PPAR*γ* may be an important signaling pathway that precipitates in WAT browning. In our mouse diabetic model and in vitro cellular model, we found that the FGFR1/FGF21/PPAR*γ* pathway was inhibited.

Recent studies indicated that miRNAs function as important regulators that participate in DM-induced atrial fibrosis [38]. The role of miR-21 in the pathogenesis of atrial fibrosis has been identified and increased miR-21 expression correlated positively with atrial fibrosis or fibrotic gene expression [18–20]. Many target genes of miR-21 have been shown to play a significant role in DM-induced atrial fibrosis via different biological pathways [39, 40]. In our previous study, we also found that miR-21 mediated a positive feedback on angiotensin II-induced myofibroblast transformation [20]. There was emerging evidence indicated that miRNAs also function as important regulators in the brown remodeling of adipocytes. Muscle-enriched miR-133a directly downregulated expression of the key transcriptional activator of brown fat differentiation, positive regulatory domain containing 16 [41]. Brown adipocyte-enriched miR-155 was also shown to inhibit brown fat cell differentiation by directly targeting the browning transcription factor, C/EBP [42]. However, it is not known whether miR-21 plays a role in the adipose browning in diabetes, thus affecting atrial fibrosis. Interestingly, in the present study, we found that the change of miR-21-3p was inconsistent with miR-21-5p in the STZ-induced mice model, and we identified miR-21-3p as a key regulator of adipose browning in hyperglycemic conditions. Furthermore, we predicted its target genes and identified the browning transcription factor, FGFR1, as its potential target. The inconsistent expression changes of miR-21-3p and miR-21-5p may be due to posttranscriptional regulation, which involved the coregulation of RNA-binding proteins, circular RNAs, long noncoding RNAs, or other microRNAs [43]. Besides, in our mouse model, we also found that miR-21-3p was significantly increased after STZ treatment in atrial tissues, which indicates that there may be a crosstalk of miRNA between EAT and atrial tissues.

A paracrine effect of EAT on the neighboring myocardium has been proposed [9]. EAT produces inflammatory mediators that can modulate the functional and structural properties of the myocardium [44]. Our previous research also showed that PPAR*γ* agonists reduced the serum inflammatory markers in patients with diabetes [45]. In the present study, we observed that miR-21-3p mimics for adipocytes could increase the levels of inflammatory factors in the coculture model in hyperglycemic conditions. Besides, in the past two decades, many adipokines have been identified and contribute directly to the regulation of chronic inflammation, vascular tone, and cardiovascular complications [46]. Among them, FGF21 is one of the first proposed adipokines released by BAT under conditions of thermogenic activation [16, 47]. The heart is one of the potential target tissues, given the reports indicating a strong cardioprotective effect of FGF21 [48]. Direct evidence was later found (as a consequence of a study on the adenosine A2A receptor in BAT) that the FGF21 released by BAT targets the heart [49]. However, whether the knockout of miR-21 mediated activation of EAT browning could promote FGF21 secretion and whether EAT browning-derived FGF21 plays an endocrine role on atrial fibroblasts in diabetes need to be further explored.

The present study has some limitations. First, mouse adipocyte-specific miR-21-3p KO was not performed. And since the small amounts of EAT in mice, we could not extract the preadipocytes of EAT to carry out subsequent experiments in vitro. Therefore, the preadipocytes were extracted from subcutaneous adipose tissue of miR-21 KO mice for further verification. Second, we only determined the extent of atrial fibrosis and did not perform the experiments of cardiac electrophysiology and programmed stimulation, such as the inducibility of AF. Finally, the STZ-induced diabetes model cannot fully reflect the physiological state of diabetes; the results need to be verified through a diet-induced obesity model in a large animal model in the future.

## 5. Conclusions

**Figure 6:**
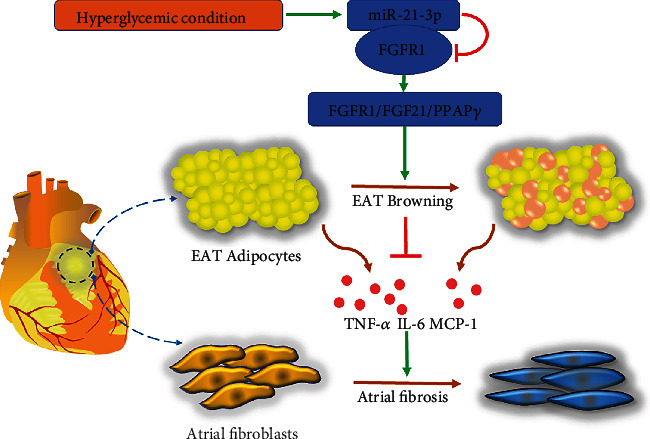
Schematic representation of the mechanisms by which miR-21-3p regulates the adipose browning-associated pathway FGFR1/FGF21/PPAR*γ* to promote HG-induced myocardial fibrosis by targeting FGFR1.

The present study shows that under hyperglycemic conditions, miR-21-3p acts as an inhibitor of adipose browning and participates in the process of atrial fibrosis. Mechanistically, downregulation of miR-21-3p increased FGFR1 expression, contributing to the activation of the browning transcriptional program via the FGFR1/FGF21/PPAR*γ* pathway (Figure 6).

## Data Availability

The data used to support the findings of this study are available from the corresponding author upon request.

## Supplementary Materials

Supplementary Materials Supplemental Table 1: the blood glucose levels of mice in each group. Supplemental Table 2: primers for real-time PCR detection. Supplemental Figure 1: the expression of different miR-21 subtypes in EAT and atrial tissues were analyzed 3 months after the modeling. (a) The relative miR-21-3p and miR-21-5p expression were detected by qRT-PCR in collected EAT. (b) The relative miR-21-3p and miR-21-5p expression were detected by qRT-PCR in atrial tissues. The data are presented as the mean ± SD of three independent experiments. ^∗∗∗^*P* < 0.001.
