# Supplementary material for: MiR-21-3p Inhibits Adipose Browning by Targeting FGFR1 and Aggravates Atrial Fibrosis in Diabetes
Source: Oxid Med Cell Longev. 2021 Aug 25;2021:9987219. doi: 10.1155/2021/9987219 (PMC8413063; doi:10.1155/2021/9987219)
Supplement: Supplementary Materials — Supplemental Table 1: the blood glucose levels of mice in each group. Supplemental Table 2: primers for real-time PCR detection. Supplemental Figure 1: the expression of different miR-21 subtypes in EAT and atrial tissues were analyzed 3 months after the modeling. (a) The relative miR-21-3p and miR-21-5p expression were detected by qRT-PCR in collected EAT. (b) The relative miR-21-3p and miR-21-5p expression were detected by qRT-PCR in atrial tissues. The data are presented as the mean ± SD of three independent experiments. ∗∗∗P < 0.001. [file 9987219.f1.docx]

**Supplementary data**

**MiR-21-3p inhibits adipose browning by targeting FGFR1 and aggravates atrial fibrosis in diabetes**

Jian-an Pan, Hao Lin, Jian-ying Yu, Hui-li Zhang, Jun-feng Zhang*, Chang-qian Wang*, Jun Gu *

Department of Cardiology, Shanghai Ninth People’s Hospital, Shanghai Jiaotong University School of Medicine, Shanghai, People's Republic of China

JAP and HL made equal contributions to this work.

**Supplemental table1.** The blood glucose levels of mice in each group**.**

| Group | ^Saline-treated WT group^ | | ^STZ-treated WT group^ | ^Saline-treated miR-21 KO group^ | ^STZ-treated miR-21 KO group^ |
| --- | --- | --- | --- | --- | --- |
| Mouse1 | | ^5.7^ | ^17.0^ | ^7.1^ | ^23.9^ |
| Mouse2 | | ^5.9^ | ^22.9^ | ^5.9^ | ^21.1^ |
| Mouse3 | | ^6.3^ | ^17.8^ | ^5.7^ | ^17.8^ |
| Mouse4 | | ^5.1^ | ^21.0^ | ^5.2^ | ^19.7^ |
| Mouse5 | | ^6.7^ | ^18.4^ | ^6.4^ | ^19.6^ |
| Mouse6 | | ^7.2^ | ^19.7^ | ^8.1^ | ^22.4^ |
| Mean±SD | | ^6.15±0.75^ | ^19.47±2.20^ | ^6.40±1.05^ | ^20.75±2.19^ |

**Supplemental table2. Primers for real-time PCR detection.**

| **Primers** | **Sequences 5'---3'** |
| --- | --- |
| FGFR1-M-Forward Primer | GGCCATCGGGCTGGATAAG |
| FGFR2-M-Reverse Primer | TGGGGACAGGGTTGGTAG |
| UCP1-M-Forward Primer | TGGCTGTTCCCACTTACTGCAC |
| UCP1-M-Reverse Primer | AGGGTCCGTCAGCATGACTC |
| GAPDH-M-Forward Primer | AGGTCGGTGTGAACGGATTTG |
| GAPDH-M-Reverse Primer | TGTAGACCATGTAGTTGAGGTCA |

The primers for miR-21-3p, miR-21-5p, cel-miR-39, U6 small nuclear RNA were obtained from RioboBio Company (Guangzhou, China). The sequences are covered by a patent.


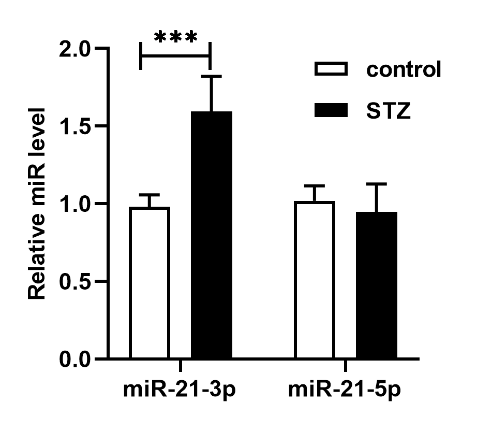

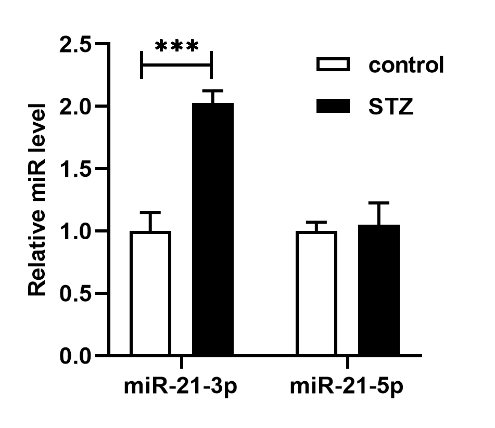
 A B

**Supplemental Figure1. The expression of different miR-21 subtypes in EAT and atrial tissues were analyzed 3 months after the modeling. A** The relative miR-21-3p and miR-21-5p expression were detected by qRT-PCR in collected EAT. **B** The relative miR-21-3p and miR-21-5p expression were detected by qRT-PCR in atrial tissues.
